# Fasting Whole-Body Energy Homeostasis and Hepatic Energy Metabolism in Nondiabetic Humans with Fatty Liver

**DOI:** 10.1155/2019/9796175

**Published:** 2019-04-11

**Authors:** Guido Lattuada, Maria Grazia Radaelli, Francesco De Cobelli, Antonio Esposito, Giuseppina Manzoni, Silvia Perra, Alessandro Del Maschio, Giovanna Castoldi, Gianluca Perseghin

**Affiliations:** ^1^Department of Medicine and Rehabilitation, Policlinico di Monza, Italy; ^2^Diagnostic Radiology San Raffaele Scientific Institute, Milan, Italy; ^3^Università San Raffaele Vita e Salute, Milan, Italy; ^4^Department of Medicine and Surgery, Università degli Studi di Milano-Bicocca, Monza, Italy

## Abstract

**Background:**

Fatty liver is believed to be sustained by a higher than normal adipose-derived NEFA flux to the liver. Also, hepatic energy metabolism may be a rate-limiting step of intrahepatic fat (IHF) accumulation.

**Aims:**

To assess whole-body energy metabolism and hepatic high-energy phosphates (HEPs) in individuals with fatty liver.

**Methods:**

We studied 22 individuals with fatty liver and 22 control individuals matched for anthropometric features by means of (1) hepatic ^1^H-magnetic resonance spectroscopy (MRS) to measure the IHF content, (2) hepatic ^31^P-MRS to assess the relative content of HEPs (phosphomonoesters, phosphodiesters, inorganic phosphorus, and ATP), and (3) indirect calorimetry to assess whole-body resting energy expenditure and substrate oxidation.

**Results:**

Patients with newly diagnosed fatty liver and controls were not different for anthropometric parameters. Based on HOMA2%-S, individuals with fatty liver were more insulin resistant than controls. Resting energy expenditure and the pattern of substrate oxidation were not different between groups. Relative content of HEPs was not different between groups; in particular, the Pi/*γ*-ATP ratio, the most important signals in terms of monitoring energy homeostasis, was not different even if it was associated with indirect calorimetry-derived parameters of oxidative substrate disposal.

**Conclusions:**

These data demonstrate that fasting whole-body energy metabolism and the relative content of HEPs in nondiabetic patients with fatty liver are not different than those in controls when they are matched for anthropometric features.

## 1. Introduction

Fatty liver is the body composition manifestation of visceral obesity in insulin-resistant subjects [1, 2]. Intrahepatic fat accumulation is thought to be due to increased adipose-derived NEFA flux to the liver [3] as reported in the fasting state [4], during euglycemic-hyperinsulinemic clamps [4, 5] and during OGTT [6]. Insulin resistance with respect to lipolysis, therefore, plays a relevant role in patients with fatty liver. Using tracer methodologies, it was found that in patients with NAFLD 60% of liver triglycerides arises from NEFA in the fasting state [7]. In the same conditions, 26% of liver triglycerides arose from de novo lipogenesis [8], and an apparent increased contribution of de novo lipogenesis vs. NEFA reesterification was reported in patients with NAFLD [7, 8]. Based on this evidence, efforts to treat hepatic steatosis by reducing fatty acids flux through dietary and pharmacological therapy (thiazolidinediones) received strong emphasis [9, 10]. Fatty liver is associated not only with hepatic but also with whole-body insulin resistance which frequently associates with higher intramyocellular lipid contents (IMCL) [11]. IMCL accumulation is associated with impaired muscle energy metabolism [12], and also, the heart of insulin-resistant subjects with fatty liver showed impaired energy metabolism [13].

Little data on the association between fatty liver and hepatic energy metabolism are currently available; Sharma et al. [14] reported that using hepatic ^31^P-MR spectroscopy, the fasting relative content of high-energy phosphates (HEPs) was altered in obese individuals with NAFLD than in controls. In the present study, we performed hepatic ^31^P-MRS to assess the liver content of HEPs and indirect calorimetry to assess whole-body resting energy expenditure and substrate oxidation to look for alterations reflecting potential abnormalities of energy metabolism in individuals with newly found fatty liver, when compared to age and BMI-matched individuals used to rule out the effect of overweight and obesity.

## 2. Materials and Methods

### 2.1. Subjects

Study subjects were selected among a large group of otherwise healthy employees of the San Raffaele Scientific Institute previously studied to assess the relationship between habitual physical activity and hepatic triglyceride content using hepatic ^1^H-MRS [15]. Within that original population, twenty-two individuals known to have excessive IHF content and twenty-two control individuals known to have normal IHF content and selected to be comparable for the anthropometric features to those with fatty liver (in order to minimize the confounding effect of obesity on the parameters of interest) performed hepatic ^31^P-MRS to assess HEP content and indirect calorimetry to assess whole-body energy metabolism. The 1 : 1 procedure of matching was based using the criteria of age within 3 years and BMI within 1 unit. This strategy has been similarly used in the past to assess whether a difference in cardiac HEP metabolism could be associated with the excessive IHF content [13], and thirteen patients out of the twenty-two with fatty liver and ten control subjects out of the twenty-two who participated to the cardiac protocol [13] performed this additional measurement of HEP content also at the level of the liver. According to the American Association for the Study of Liver Diseases (AASLD), normal or higher than normal IHF content was set at 5% ww [16]. Outpatients admitted to the Center of Nutrition/Metabolism of the San Raffaele Scientific Institute were studied. In order to be recruited, body weight had to be stable for at least six months. Patients with history of hepatic disease, substance abuse, or daily consumption of more than one alcohol drink daily (<20 g/day) or the equivalent in beer and wine were excluded from the study.  Table 1 summarized the anthropometric characteristic of the recruited subjects, which, based on the medical history, physical examination, blood and urinary tests, were in good health. An informed written consent was collected for each subject who participated in the study. Recruited subjects gave their informed written consent after the explanation of purposes, nature, and potential risks of the study. The Ethical Committee of the Istituto Scientifico H San Raffaele approved the studies.

### 2.2. Experimental Protocol

In three days before the study, the recruited subjects were asked to consume isocaloric diet and to abstain from exercise activity. They were studied after an 8-10-hour overnight fast by means of ^31^P-MRS and ^1^H-MRS for the assessment of the hepatic relative high-energy phosphates and the IHF content, respectively. Assessment of whole-body energy metabolism by means of indirect calorimetry and blood drawing to measure serum insulin, C-peptide, plasma glucose, NEFA, lipid profile, and biochemical parameters was performed the same morning or a few days apart.

#### 2.2.1. ^31^P-MR Spectroscopy

Hepatic ^31^P-MRS was performed with volunteers in the supine position within a 1.5 T whole-body scanner (Gyroscan Intera Master 1.5 MR System; Philips Medical Systems, Best, the Netherlands). ^31^P spectra were obtained by means of a 10 cm diameter surface coil used for the transmission and detection of radio frequency signals at the resonance frequency of ^31^P (at 1.5 T, 25.85 MHz). A small sample container built in the coil center, containing an aqueous solution of methyl-phosphonate, served as geometrical reference. The surface coil was secured in place with a Velcro band around the abdomen and chest, helping to minimize breathing artifacts. MR imaging was performed to acquire scout images, to establish the exact position of the ^31^P surface coil, and eventually to reposition it. Localized homogeneity adjustment was performed using the body coil by optimizing the ^1^H-MR spectroscopy water signal. Shim volumes were planned on the transverse and sagittal scout images. The transmitter-receiver was then switched without time delay to the ^31^P frequency. Manual tuning and matching of the ^31^P surface coil was performed to adjust for different coil loading. The radio frequency level was adjusted to obtain a 180° pulse of 40 ms for the reference sample at the center of the ^31^P-surface coil. The acquisition of ^31^P-MR spectra was performed with a recycle time of 3.6 s. ISIS volume selection in three dimensions (3D-ISIS) was employed. It was based on 192 averaged free induction decays. The Volume of Interest (VOI) was oriented perpendicular to the abdomen wall, avoiding inclusion of abdominal wall muscle and diaphragm muscle. The volume size was approximately 3 (caudocranial) × 4 × 4 cm^3^. The acquisition time was 11 min. Adiabatic frequency-modulated hyperbolic secant pulses and adiabatic half-passage detection pulses were used to achieve inversion and excitation over the entire VOI. The examination time was 40-45 min. 3D-ISIS was employed after testing that uses higher spatial resolution (2D-ISIS+1D SI using a one-dimensional phase encoding bar with 32 rows of 1 cm thickness each angulated perpendicular to the abdominal wall for the anterior-posterior direction and lateral and craniocaudal dimensions dependent on the patients' liver size); the Pi/*γ*-ATP ratios were in agreement and showed absence of muscle PCr signal.

#### 2.2.2. ^1^H-MR Spectroscopy

Hepatic ^1^H-MR spectroscopy was performed in all volunteers with the same MR system as previously described [15].

#### 2.2.3. Indirect Calorimetry

After lying quietly for 30 min, REE was measured by continuous indirect calorimetry with a ventilated hood system (SensorMedics 2900, Metabolic Measurement Cart) performed for 45 min as previously described [17]. The mean coefficient of variation (CV) within the session for both O_2_ (2.1 ± 0.2%) and CO_2_ (2.3 ± 0.3%) measurements was below 5%.

### 2.3. Analytical Determinations

Glucose (Beckman Coulter Inc., Fullerton, CA), FFA, triglycerides, total cholesterol, and HDL cholesterol were measured as previously described [15]. Plasma levels of insulin (sensitivity 2 *μ*U/ml; intra- and interassay CV <3.1% and 6%, respectively) were measured with RIA (Linco Research, Missouri, USA). Plasma C-peptide was measured (CVs: intra − assay = 2.3%, interassay = 4.1%) using a double-antibody RIA kit (Diagnostic Product Corporation, Los Angeles, CA).

### 2.4. Calculations

**Figure 1:**
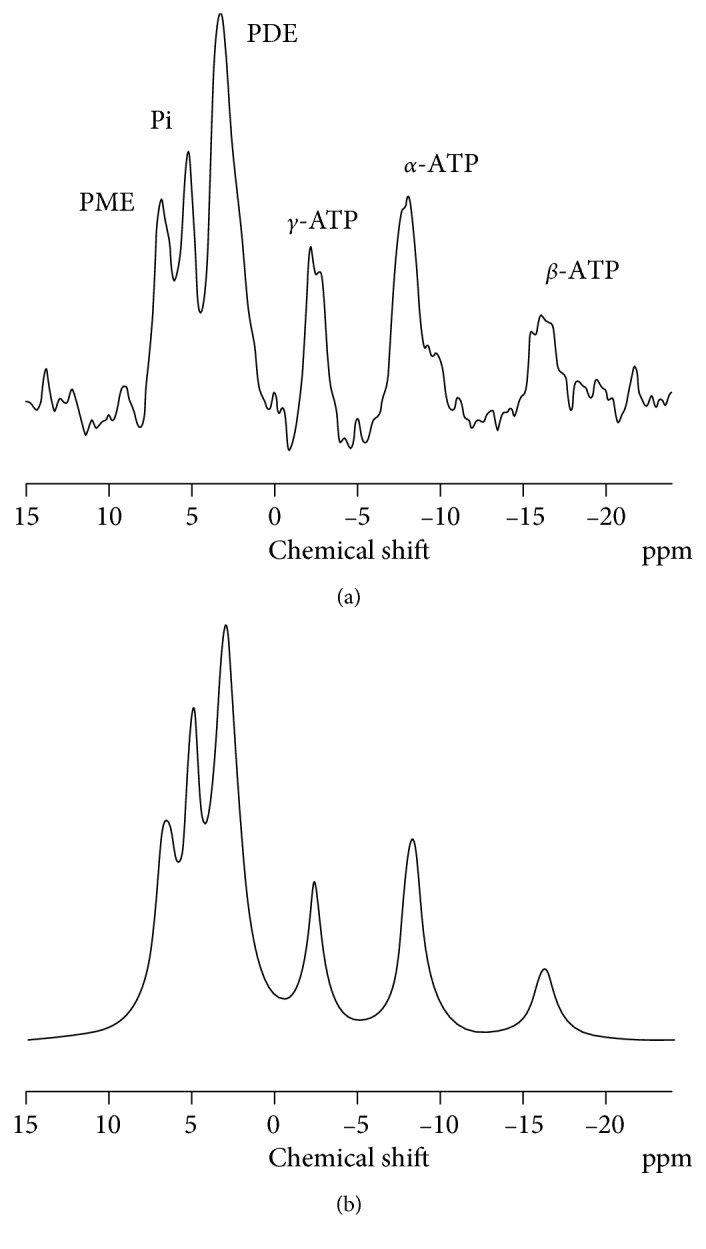
A typical ^31^P spectrum of a normal subject is depicted in (a). In (b), the spectrum after line-fitting procedures is shown. The signals that can be detected are the signals of the three phosphorous atoms of adenosine triphosphates (*γ*, *α*, and *β*). Phosphomonoesters (PME), inorganic phosphorous (Pi), and phosphodiesters (PDE) are detectable at ppm on the left side of the spectrum. PMEs (phosphocholine, phosphoethanolamine, adenosine monophosphate, and glycolytic intermediates including glucose-6-phosphate) and PDEs (glycerolphosphorylcholine and glycerolphosphorylethanolamine) represent a heterogeneous mix of compounds that share only a similar chemical feature but otherwise have diverse chemical structures and functions. Pi signal corresponds to free inorganic phosphorous. In contrast with the skeletal muscle and the heart, very little, if any, phosphocreatine is detectable in the liver (chemical shift at 0 ppm); its presence in the spectrum is likely a marker of extrahepatic muscle contamination from malpositioning of the spectroscopic voxel.

Fasting-based indices of insulin sensitivity (HOMA2-%S) and *β*-cell responsivity (HOMA2-%B) were determined by the updated HOMA2 method [18] available from http://www.OCDEM.ox.ac.uk. ^31^P-MR spectra were transferred to a remote SUN-SPARC workstation for analysis. The spectra were quantified automatically by model function analysis in the time domain. The spectral fitting routine was based on a nonlinear least-squares Gauss-Newton implementation for exponential damping as previously performed for the postprocessing of spectra obtained from transplanted kidney [19]. A typical ^31^P spectrum is depicted in Figure 1 with description of the signals in the legend. *γ*-ATP and Pi are considered the most important signals in terms of monitoring energy homeostasis because the *γ*-ATP phosphorous is the one which is released as Pi in the reaction of ATP hydrolysis to ADP. For this reason, in the present work, we have chosen the Pi/*γ*-ATP and the Pi/ATP (mean of the three phosphorous signals) ratios as markers of intrahepatic ATP metabolism. We also calculated the PME/Pi, PME/*γ*-ATP, and PME/ATP ratios as comparison with previously published data in hepatic diseases. The percent IHF was calculated as previously described [14]. REE was calculated by Weir's standard equation [20] from the O_2_ consumption rate and the CO_2_ production rates measured by means of indirect calorimetry (excluding the first 10 min of data acquisition) and from the urinary nitrogen excretion. Predicted REE was calculated using the Harris-Benedict equations [21]. Glucose, lipid, and protein oxidation was estimated as previously described [22].

### 2.5. Statistical Analysis

Data in text tables are means ± SD. Analyses were performed using the SPSS software (ver. 13.0; SPSS Inc., Chicago). Comparison between groups was performed using the 2-tailed independent-samples *t*-test, and a *P* value less than 0.05 was considered to be significant. Variables with skewed distribution assessed using the Kolmogorov-Smirnov test of normality were log-transformed before the analysis. Correlation analysis was performed using two-tailed Pearson's correlation.

Sample size calculation was based on preliminary acquisitions in a small (*n* = 12) group of normal-weight subjects. 11 individuals for each group provided a power of 85% using a *t*-test at a one-side *α* of 0.05 when assuming a 20% difference between groups in the Pi/*γ*-ATP ratio with a standard deviation of 0.50.

## 3. Results

### 3.1. Anthropometric and Laboratory Characteristics of Study Subjects

Individuals with or without fatty liver (mean ± standard deviation and ranges of the IHF content are summarized in Table 1) were not different with respect to age and BMI (Table 1). Subjects with excessive IHF content had higher serum triglycerides and plasma C-peptide concentrations. HOMA2-S, as a surrogate index of insulin sensitivity, was lower in subjects with fatty liver than in normals.

### 3.2. Parameters of Whole-Body Energy Metabolism

Resting energy expenditure (REE) and the percent of predicted REE were not different between groups. Also, whole-body glucose and lipid oxidation was not different between groups as reflected by the similar respiratory quotient (Table 2).

### 3.3. Intrahepatic High-Energy Phosphates

The intrahepatic high-energy phosphate content is summarized in Table 2. The peak area of each metabolite (PME, Pi, PDE, *γ*-ATP, *α*-ATP, and *β*-ATP) was expressed as a percentage of the total ^31^P-MR signal (the sum of all the resonances). No difference was detected for each of the metabolites between the two groups of study. Also, the Pi/*γ*-ATP, Pi/ATP, PME/Pi, PME/*γ*-ATP, and PME/ATP ratios were not different between groups.

### 3.4. Correlation Analysis with Parameters of Hepatic High-Energy Phosphates

The Pi/*γ*-ATP ratio was not associated with anthropometric features or with the features of the metabolic syndrome. It was not associated with the IHF content (*r* = −0.001; *P* = 0.96). It was not associated with parameters of lipid metabolism and fasting plasma NEFA or with fasting plasma glucose, insulin, and HOMA indices. Interestingly, it was associated with indirect calorimetry-derived parameters of oxidative substrate disposal; in fact, it correlated with the respiratory quotient (*r* = −0.52; *P* = 0.001), the fasting whole-body glucose oxidation (*r* = −0.57; *P* = 0.001), and the fasting lipid oxidation (*r* = 0.45; *P* = 0.005). This association with the respiratory quotient was detectable in a more robust fashion in individuals with fatty liver (*r* = −0.75; *P* = 0.0001) than in the control individuals (*r* = −0.34; *P* = 0.03). A similar pattern of correlation was found when the Pi/ATP ratio was used. The PME/Pi and PME/ATP ratios were not correlated with any of the metabolic parameters summarized in Table 1.

## 4. Discussion

The present study demonstrates that in nondiabetic individuals with newly found fatty liver, the fasting whole-body energy metabolism and the relative amount of hepatic HEPs are not different than in age- and BMI-matched nondiabetic individuals without fatty liver.

Little data about whole-body energy metabolism were available in patients with fatty liver. Bugianesi et al. [23] reported that during hyperinsulinemic conditions, whole-body lipid oxidation rate was higher in a small group of individuals with biopsy proven NAFLD (*n* = 12) when compared to 6 control subjects. As discussed in the introduction section, this finding may be secondary to the higher plasma NEFA characterizing these patients [4–6]. In our study, we did not assess insulin sensitivity with the clamp procedure, but based on the higher insulin resistance estimated using the fasting-derived index (HOMA2-S in Table 1), it is likely that also our cohort of subjects in the same insulin-stimulated condition could be characterized by higher lipid oxidation rate. The other facet of energy metabolism is the postabsorptive and resting state. In our cohort of individuals with newly diagnosed fatty liver, postabsorptive whole-body energy metabolism was not different than that of controls in absolute terms and when normalized to the predicted values based on sex, age, race, body weight, and height. In addition, substrate oxidative disposal was remarkably similar to that of controls based on semiquantitative parameters such as the respiratory quotient and also based on the oxidative fluxes (Table 2). This may be substantially in agreement with the report of Bugianesi et al. showing only a trend (*P* = 0.09) for higher fasting lipid oxidation rate in their 12 patients when compared to 6 controls. It is possible that a more severe hepatic disease in Bugianesi's cohort may explain this little discrepancy. In fact, we have also observed that in more serious hepatic diseases, such as in individuals with cirrhosis and hepatocarcinoma, a hypercatabolic state sustained by higher fasting lipid oxidation rate could be detected and that liver transplantation in the long-term normalized this abnormality [17].

We may conclude that in individuals with newly diagnosed fatty liver, whole-body energy metabolism is not different than in age- and BMI-matched individuals during the postabsorptive and resting state.

With respect to the local hepatic energy metabolism as assessed by means of ^31^P-MRS, Sharma et al. [14] reported higher PME/Pi, PME/*γ*-ATP, PME/*β*-ATP, and PME/ATP in overweight/obese Asian Indians with NAFLD when compared with normal weight individuals, either with or without NAFLD. We believe that the different HEP contents were not due to the fatty liver per se but rather to the different degrees of obesity. Several observation may support our thinking. In our study, when we compared the overweight subjects with fatty liver with a group of similarly overweight subjects without fatty liver, a difference was not detectable (Table 2). Taking a careful look to the data by Sharma et al. [14], it may be observed that when anthropometric parameters were matched between individuals with and without NAFLD (in their study the comparison may be performed at a normal range of BMI: 22 kg/m^2^), the difference was not significant also in their own set of data. In support of this hypothesis, a pilot study using ^31^P-MRS of the liver suggested that in 8 obese individuals with NASH, ATP recovery within the liver after the administration of fructose e.v. was severely blunted, but the authors reported that the recovery became progressively less efficient as BMI increased also in the group of healthy controls [24]. This study would also confirm that this impairment is present in association with obesity per se, and this suggestion is also given by another study in which ATP was monitored in normal weight and obese individuals in the fasting state and after fructose administration [25].

An alternative potential explanation for this controversial finding is related to a limitation of our and Sharma's works. In both studies, there was a lack of histological characterization of the hepatic disease; therefore, no information about the inflammatory status, necrosis, and fibrosis was available. It may not be excluded that different histological features of the disease may be able to explain the reported differences in HEP relative content. In fact, increased PME ratios have been repeatedly found in patients with more severe liver diseases including chronic hepatitis C [26], liver cirrhosis [27], or tumors [28], all conditions in which extensive membrane remodeling may have an effect on the membrane phosphocholine and phosphoethanolamine kinetics and ultimately in PME content.

Based on our own data, we therefore would like to conclude that the relative content of hepatic HEPs is not different in individuals with newly found fatty liver that in controls when study groups are comparable for body mass and that the previously reported differences may be secondary to body fatness rather than fatty liver per se.

In summary, our set of data would support the hypothesis that fasting whole-body and hepatic energy metabolism is not abnormal in individuals with fatty liver when compared to matched controls in agreement with findings generated using different methodological tools [29], in contrast with the finding within the skeletal muscle [12] and the heart of insulin-resistant individuals [13].

Since in the postabsorptive and resting state the liver, along with the skeletal muscle, is the tissue contributing most to energy expenditure, we wondered whether the acquired parameters could be somehow related to each other. We noticed that a significant correlation could be detected between the whole-body substrate oxidative disposal (expressed as respiratory quotient, glucose, and lipid oxidation) and the Pi/*γ*-ATP ratio. The correlation would imply that a relatively higher hepatic content of Pi (a more catabolic condition if the hepatocellular ATP content is postulated to be comparable) is present when lipids are the preferential substrate for the oxidative processes. This correlation was not a peculiar feature of the individuals with fatty liver but was detectable also in the control individuals without fatty liver even if by a minor extent.

Even if these ^31^P-MRS studies do not allow the assessment of the absolute hepatic HEP concentrations, which represents a methodological limitation, the expression of the signal intensities as ratios would imply that the intracellular hepatic ATP concentration is constant and not different between groups. A higher Pi/*γ*-ATP ratio would therefore imply for a catabolic energetic state of the hepatocytes, in which a reduced phosphorylation status would be reflected by an increase of Pi levels relative to ATP. The lack of difference of the Pi/*γ*-ATP ratio between groups we are reporting in the present work would therefore imply a normal hepatic energy metabolism, even if we cannot exclude that the absolute Pi and ATP hepatocellular content may be consensually higher and/or lower in parallel resulting in no change in the ratio. We believe that the lack of differences in the Pi/*γ*-ATP ratio between the groups in the present work implies a normal hepatic metabolism because we also analyzed our data as the relative percent contribution of each single signal to the total ^31^P detectable signals, as summarized in Table 2, and once more we found no significant difference between groups. It is important to emphasize that future studies will require the determination of the absolute substrate concentration. With this respect, Chmelík et al. [30] have validated a spectroscopic absolute quantification of ^31^P metabolites in human livers based on the use of an external phantom with known concentrations of the substrates of interest.

In conclusion, the present work demonstrates that fasting and resting whole-body energy metabolism and the relative content of HEPs in nondiabetic patients with newly diagnosed fatty liver are not different than those in controls when they are matched for anthropometric features. Absolute quantification of these substrates remains a mandatory step to firmly establish whether alteration of hepatic energy metabolism is present in individuals with fatty liver.

## Figures and Tables

**Table 1:** Biochemistry and clinical features of individuals with newly diagnosed fatty liver and controls.

	Fatty liver	Controls	*P* value
Number and sex (F/M)	22 (2/20)	22 (2/20)	
Age (years)	34 ± 7	35 ± 8	
BMI (kg/m^2^)	27.9 ± 2.9	27.7 ± 1.8	
Total cholesterol (mg/dl)	195 ± 39	185 ± 32	0.37
HDL cholesterol (mg/dl)	45 ± 12	52 ± 13	0.07
Triglycerides (mg/dl)	169 ± 85	91 ± 42	0.01
NEFA (mg/dl)	0.61 ± 0.18	0.60 ± 0.29	0.91
Creatinine (mg/dl)	0.85 ± 0.22	0.94 ± 0.14	0.29
Plasma glucose (mg/dl)	91 ± 9	89 ± 11	0.62
Plasma insulin (*μ*U/ml)	17 ± 6	11 ± 4	0.06
Plasma C-peptide (ng/ml)	3.20 ± 1.21	2.29 ± 0.82	0.01
HOMA2-B (%)	160 ± 39	149 ± 53	0.46
HOMA2-S (%)	51 ± 14	72 ± 51	0.05
IHF content (% wet weight)	13.93 ± 8.25	2.43 ± 0.99	0.0001
	Range: 5.40–39.25	Range: 0.84–4.28	

Mean ± SD; independent-samples *t*-test (2-tailed). HOMA: HOMA2: homeostatic model assessment; HOMA2-S: insulin sensitivity; HOMA2-B: *β*-cell sensitivity.

**Table 2:** Whole-body energy metabolism as assessed by indirect calorimetry and high-energy phosphate hepatic relative content as assessed by ^31^P MRS.

	Fatty liver	Controls	*P* value
Indirect calorimetry			
REE (Kcal/die)	1935 ± 368	1886 ± 261	0.64
% of predicted REE (%)	107 ± 12	103 ± 12	0.25
Respiratory quotient	0.81 ± 0.05	0.81 ± 0.05	0.93
Glucose oxidation (mg/(kg min))	1.30 ± 0.74	1.25 ± 0.75	0.83
Lipid oxidation (mg/(kg min))	0.93 ± 0.36	0.87 ± 0.35	0.58
Hepatic ^31^P-MRS			
% PME	12 ± 5	11 ± 5	0.48
% Pi	11 ± 4	11 ± 4	0.94
% PDE	42 ± 6	41 ± 6	0.72
% *γ*-ATP	10 ± 2	10 ± 2	0.50
% *α*-ATP	17 ± 3	19 ± 3	0.10
% *β*-ATP	7 ± 3	8 ± 2	0.47
% ATP	11 ± 2	12 ± 2	0.49
Pi/*γ*-ATP ratio	1.17 ± 0.55	1.19 ± 0.34	0.83
Pi/ATP ratio	1.00 ± 0.46	0.95 ± 0.27	0.63
PME/Pi ratio	1.14 ± 0.50	1.04 ± 0.57	0.56
PME/*γ*-ATP ratio	1.30 ± 0.72	1.24 ± 079	0.79
PME/ATP ratio	1.14 ± 0.67	0.96 ± 0.55	0.36

The peak area of each metabolite (PME, Pi, PDE, *γ*-ATP, *α*-ATP, and *β*-ATP) was expressed as a percentage of the total ^31^P-MR signal (the sum of all the resonances). Mean ± SD; independent-samples *t*-test (2-tailed). REE: resting energy expenditure.

## Data Availability

The data used to support the findings of this study are available from the corresponding author upon request.

## Abbreviations

^31^P-MRS: Phosphorus-31 magnetic resonance spectroscopy
^1^H-MRS: Proton magnetic resonance spectroscopy
IHF: Intrahepatic fat
HEPs: High-energy phosphates
NEFA: Nonesterified fatty acids
PME: Phosphomonoesters
Pi: Inorganic phosphorous
PDE: Phosphodiesters
PCr: Phosphocreatine
OGTT: Oral glucose tolerance test
BMI: Body mass index
3D-ISIS: ISIS volume selection in three dimensions
REE: Resting energy expenditure
RQ: Respiratory quotient
HOMA: Homeostatic model assessment
IMCL: Intramyocellular lipid contents
NAFLD: Nonalcoholic fatty liver disease.
